# Evaluation of morpho-physiological responses and genotoxicity in *Eruca sativa* (Mill.) grown in hydroponics from seeds exposed to X-rays

**DOI:** 10.7717/peerj.15281

**Published:** 2023-04-26

**Authors:** Maria Cristina Sorrentino, Angelo Granata, Mariagabriella Pugliese, Lorenzo Manti, Simonetta Giordano, Fiore Capozzi, Valeria Spagnuolo

**Affiliations:** 1 Department of Biology, University of Naples Federico II, Naples, Italy; 2 Department of Physics, University of Naples Federico II, Naples, Italy

**Keywords:** Plants and abiotic stress, Ionizing radiation, ISSR markers, Antioxidant, Rubisco, Effects on stomata

## Abstract

Due to its potential applications in cultivated plants, ionizing radiation (IR) and its effect on organisms is increasingly studied. Here we measured the effects of ionizing radiation on *Eruca sativa* by analyzing plants from irradiated seeds (1 and 10 Gy) grown in hydroponics. We measured several morpho-physiological traits and genotoxicity. Radiation stress induced a noticeable variability of the morpho-physiological traits highlighting decreased plant vigor. Shoot length and leaf number were significantly higher in 1 Gy-treated samples, whereas root length was significantly higher in 10 Gy treated plants. Stomata number significantly increased with IR dose, whereas both pigment and Rubisco content decreased under radiation stress. Phenol content significantly increased in 1 Gy treated samples, otherwise from total antioxidants, which were not different from control. Most results could find a feasible explanation in a hormesis-like pattern and in a decreased plant vigor under radiation stress. IR induced genotoxic damage, evaluated by ISSR markers, in 15 day old leaves; specifically, a severe decrease in the genome template stability was observed. However, a partial recovery occurred after 2 weeks, especially under the lowest dose (*i.e*., 1 Gy), suggesting that DNA damage detection and repair mechanisms are active. Pigment content and genotoxic damage may serve as proxies for evaluating plant responses to IR stress, since they show univocal dose-dependent trends. The use of more checkpoints for analyses and more doses over a wider range, as well as the focus on different metabolites, could help elucidate plant response in terms of morpho-physiological changes.

## Introduction

Ionizing radiation (IR) and its effect on living organisms is increasingly investigated, both because it represents a major issue for advances in space exploration, and for its potential applications in cultivated plants (Couronne, 2019; Dreier, 2016; Leniart, 2017). For instance, IR has been recently shown to increase the concentration of bioactive substances, such as antioxidants, extracted from plant waste (Madureira et al., 2020). IR, such as gamma rays, X-rays, and higher UV radiation, have enough energy to ionize atoms or molecules (Friedberg & Copeland, 2011; Woodwell, 1962). They may have a direct effect when hitting a target molecule, or an indirect effect when determining ROS production (Esnault, Legue & Chenal, 2010; Gudkov et al., 2019). Many ordinary activities expose humans to small doses of IR, such as medical imaging, an airplane trip, and of course the natural radiation background, also affecting all living organisms, included plants. The effects of IR on organisms can be dose-dependent and related to the exposure duration; they may involve both morphological traits and physiological pathways. In plants, IR inhibits growth rates, reducing biomass production, because of the decline in photosynthesis (*e.g*., Arena et al., 2013) IR can also affect gene expression and cell reproduction (Esnault, Legue & Chenal, 2010). Furthermore, IR induces genetic damage by altering DNA structure (Kim et al., 2019); these alterations can be easily assessed in term of genome template stability (GTS) by multilocus molecular markers, such as inter simple sequence repeats (ISSR), already tested to highlight the genetic damage under X-rays stress in plants (Sokolova et al., 2022). In parallel, IR activates several compensative responses, like the production of ROS scavengers and the increase of antioxidant compounds (Madureira et al., 2020).

It is also reported that low doses of IR may determine beneficial responses, although this is still a quite controversial issue (Real et al., 2004; Vanhoudt et al., 2010). The beneficial effect of low doses of IR fits the definition of hormesis (de Sousa Araújo et al., 2016). Hormesis is indeed an adaptive response of cells and organisms to moderate stresses, through the activation of cellular defense and repair mechanisms against stressors (Mark, 2008). IR represent a resource in agricultural sciences and food technology since they are used at low doses to improve food safety and storability. Among the IR indeed, γ-rays are mainly used in high sterilization protocols (Jayawardena & Peiris, 1988) and have been studied in deep in relation to their ability as pre-sowing invigoration treatments in seed technology (de Sousa Araújo et al., 2016). X-rays can increase food safety and storability (Farkas & Mohácsi-Farkas, 2011), but its long-term effects on plants is unclear (de Sousa Araújo et al., 2016). Recent studies focused on X-rays provided contrasting information on their possible beneficial or harmful role and indicate that the effects observed are dose and specie-specific (De Micco et al., 2014; Esnault, Legue & Chenal, 2010). For example, X-rays are known to reduce seed germination percentage and root growth of *Phoenix dactylifera* L., but, a stimulatory effect on leaf growth was observed when seeds were irradiated with a 0.65 Gy dose (Al-Enezi, Al-Bahrany & Al-Khayri, 2012). In addition, seed irradiation of *Solanum lycopersicon* L. stimulated growth parameters, especially at 10 Gy treatment (De Micco et al., 2014). Plant responses to low doses (*i.e*., up to 10 Gy) is also interesting for space-related issues, since it has been estimated that a permanence of 1 year in Space would expose organisms to a dose of less than 10 Gy (Bennett, Pirim & Orlando, 2013; De Micco et al., 2014; Kerr, 2013).

*Eruca sativa* Mill. is a member of the Brassicaceae, and thanks to its organoleptic properties, fiber content and relative ease of cultivation can be also considered a good candidate as a future space food (Dueck et al., 2016; Rivera et al., 2006). Under the hypothesis that ionizing radiations can affect morphophysiological traits, including DNA, the aim of this work was to evaluate the effects induced by 1 and 10 Gy X-ray irradiation in *Eruca sativa*. The biological traits measured were: (i) morphological growth parameters; (ii) stomata size and number; (iii) pigment and Rubisco contents; (iv) total antioxidants and phenols; (v) genotoxicity, evaluated at two different growth times to estimate a possible recovery.

## Materials and Methods

### Seed irradiation and plant culture condition

Seeds of *Eruca sativa* were irradiated by 1 and 10 Gy X-rays at the Department of Physics (University of Napoli Federico II, Napoli, Italy) by means of a radiogenic tube (STABILIPAN II; Siemens, Berlin, Germany) at the Radiation Biophysics Laboratory. Doses were administered through a 1-mm Cu filtration at a dose rate of about 1.36 Gy min^−1^. Dosimetry checks were routinely performed by an ionization chamber to ensure dose uniformity within a square field of 15 cm side. Irradiated seeds and non-irradiated seeds used as control were germinated on wet filter paper in the dark at 25 °C for 4 days. When the roots emerged and the cotyledons appeared fully developed, 15 seedlings for each treatment and control were transplanted to a hydroponic system (Sorrentino et al., 2021) containing 5 L of nutrient solution (Hoagland & Arnon, 1950) at pH 5.8 (Fig. S1). The growth chamber was settled at the following conditions: (1) temperature 24/18 °C; (2) relative humidity (RH) 55–75% (day/night); (3) photoperiod of 16 h light per day with a photosynthetic photon flux density (PPFD) at the top of the canopy of 180–190 μmol photons m^−2^ s^−1^. All plants were analyzed after 30 days culture.

### Morphological traits, stomata counting and sizing

Length and weight of the root and shoot, and leaf number were considered in 10 plants after 30 days culture for each treatment and control for morphometrical analysis. Stomata number was determined on five fully expanded leaves for each treatment. Two surface replicas were obtained by nail topcoat at midlamina of the leaves. For each treatment 10 surface areas of about 160,000 µm^2^ were observed under a light microscope (for a total analyzed surface of 1.6 mm^2^ per treatment) and images acquired were analyzed by ImageJ software (National Institute of Health, Bethesda, MD, USA).

### Photosynthetic pigment content

Chlorophyll a, chlorophyll b and carotenoid contents were determined in five leaves (from different plants) per treatment following the procedure described by Lichtenthaler (1987). In brief, pigments were extracted from fresh leaf tissue previously weighed (200–300 mg) by mortar and pestle in ice-cold 100% acetone and centrifuged at 5,000 rpm for 5 min (Labofuge GL; Heraeus Sepatech, Hanau, Germany). The absorbance of supernatants was quantified by spectrophotometer (Cary 100 UV-VIS; Agilent Technologies, Santa Clara, CA, USA) at 470, 645 and 662 nm and pigment concentrations expressed in µg g^−1^ FW, calculated as follows:

[Chl a] = 11.24 * A_662_ – 2.04 * A_645_
[Chl b] = 20.13 * A_645 −_ 4.19 * A_662_
[Chl a + Chl b] = 7.05 * A_662_ + 18.09A_645_
[c + x] = (1000 * A_470_ − 1.9 * [Chl a] − 63.14 * [Chl b])/214

where [Chl a], [Chl b] and [c + x] are the concentrations of chlorophyll a, chlorophyll b, and total carotenoids, respectively.

### Protein analysis

Photosynthetic protein analysis was carried out because protein pattern may be severely affected by abiotic stress, including radiation (*e.g*., Arena et al., 2013). Protein extraction of leaves was carried out on plants after 1-month growth using 0.3 g of plant material for each sample (Bertolde et al., 2014; Wang et al., 2006). An SDS-PAGE (10%) was performed by using Dual Color Protein Standard (Bio-Rad, Hercules, CA, USA) as marker and Laemmli loading buffer added to the samples to follow protein separation. Western blot analysis on protein samples was performed using a blocking solution (100 mM Tris-HCl pH 8.0, 150 mM NaCl, 0.1% Tween 20, 5% BSA) and primary specific antibodies (Agrisera) to reveal the Rubisco (anti-RbcL, rabbit polyclonal serum), and the Actin (anti-ACT, rabbit polyclonal serum), used as loading control. The immunorevelation was performed using the kit for chemiluminescence (Westar Supernova; Cyanagen, Bologna, Italy) by ChemiDoc System (Bio-Rad, Hercules, CA, USA). Densitometry analysis was performed following Sorrentino et al. (2018) using ImageJ software (Rasband, W.S., U.S. NIH, Bethesda, MD, USA, 1997–2012), normalizing each Rubisco band value to the corresponding actin band value. Results were expressed as percentages of the control set to 100%.

### Antioxidants

The antioxidant analysis was carried out by the ferric reducing antioxidant power assay (FRAP). Powdered fresh leaf samples 250 mg each (homogenized in a TissueLyser LT after freezing in liquid nitrogen), were mixed with 60:40 (v/v) methanol/water solution and centrifuged at 14,000 rpm for 15 min at 4 °C. Then, acetate buffer was added to the extract (1:16 300 mM pH 3.6); The acetate buffer contained a mix of tripyridyltriazine (TPTZ) (10 mM TPTZ in 40 mM HCl, 1:1.6) and FeCl_3_ (1:16 12 mM FeCl_3_). After 1 h of incubation at 4 °C, the absorbance was measured at 593 nm with a spectrophotometer (UV-VIS Cary 100; Agilent Technologies, Palo Alto, CA, USA) using Trolox (6-hydroxy-2,5,7,8-tetramethylchroman-2-carboxylic acid) as standard. The antioxidant capacity was expressed as μmol Trolox equivalents for mg of fresh sample.

### Phenols

The assay by Folin–Ciocalteu (F–C) reagent was used to quantify the total phenolic compounds (Singleton & Rossi, 1965). The F–C assay is based on the oxidation of phenols in alkaline environment, with the transfer of electrons to phosphomolybdic/phosphotungstic acid complexes, whose reduced form appears blue. Phenolic concentration was determined by a spectroscopy at 760 nm. Although the electron transfer reaction is not specific for phenolic compounds, the extraction procedure eliminates approximately 85% of ascorbic acid and other potentially interfering compounds (Ainsworth & Gillespie, 2007).

In brief, 1 ml of 60% methanol was added to 250 mg of ground fresh tissue (homogenized in a TissueLyser LT after freezing in liquid nitrogen) in a 1.5 ml tube. The samples were mixed by vortexing and placed on ice for 3 min in the dark and then transferred into a 15 ml tube; the volume was brought to 5 ml with the addition of 60% methanol. The samples were then centrifuged at 3,000g for 5 min. Then 62.5 μl of supernatant were added to 62.5 μl of Folin-Ciocalteu’s reagent (Sigma, St. Louis, MI, USA) and 250 μl of deionized water and incubated for 6 min at room temperature. After the addition of 625 μl of sodium carbonate 7.5% and 500 μl of deionized water, the samples were incubated for 90 min at room temperature. The absorbance was measured at 760 nm.

Total phenolic was expressed in μg of gallic acid equivalent/mg of fresh weight, based on the standard curve of gallic acid drawn on a dynamic range between 0 and 125 μg of gallic acid.

### Genotoxicity

Three young leaves (15 d), at the same development stage, were selected from three plants for each treatment (control, 1 and 10 Gy X-ray irradiation), and DNA-extracted and amplified following Sorrentino et al. (2022). In brief, total genomic DNA was extracted by using a modified CTAB (cetyl-trimethyl ammonium bromide) method (Murray & Thompson, 1980). DNA amplification was performed by using six different inter simple sequence repeat (ISSR) primers (Table S1).

Polymerase chain reactions (PCRs) were carried out three times for each primer to confirm the reproducibility of banding profiles; however, only constant bands were selected for the analysis and Genome template stability (GTS) calculation. Each amplification reaction contained DNA (15 ng), ddH_2_O, 10X Dream Taq Buffer, 1 mM MgCl_2_, 0.2 mM each dNTP, 0.6 mM primer, and 1 U Dream Taq DNA polymerase in a final volume of 25 µL (Thermo Fisher Scientific, Waltham, MA, USA).

The thermocycler (Esco Swift Maxi Thermal Cycler) was programmed for an initial denaturation step at 94 °C for 3 min, followed by 35 cycles with 1 min at 94 °C, 1 min annealing and 2 min extension, and a final extension cycle of 7 min at 72 °C. Amplification were run by electrophoresis on 2% agarose gel with Tris-borate-EDTA (TBE) buffer; amplification products stained with GelRed Nucleic Acid 10,000 (biotium) were visualized under UV light using GelDoc (Bio-Rad, Hercules, CA, USA). Size estimates of the ISSR bands were made using a 100-bp ladder. ISSR profiles (*i.e*., bands between 200 and 1,300 bp) for each primer were analyzed by using the software GelAnalyzer 2019 for band counting and intensity assignment. Changes in the ISSR patterns were expressed as a decrease in GTS, a quantitative measure of DNA stability based on ISSR banding of treated samples compared to the control samples. The GTS was calculated by the formula:

(1) $$\rm {GTS=[1 - a/n] \times 100}$$

where a is the average number of polymorphic bands in each treated sample and n the number of all bands in the control. Therefore, GTS values expresses the percentage of non-polymorphic bands. Firstly, genetic variation was also estimated among the three control plants to establish within-control variation as a threshold to evaluate the eventual occurrence of the genotoxic damage. Then, DNA profiles of one control plant was arbitrarily fixed as a reference control plant to be compared to DNA profiles of the plants from X-ray-treated seeds. The differences in band intensity were also calculated by GelAnalyzer software in comparison to the bands of the 100 bp ladder (here used as gel marker), having known DNA concentrations; only difference of intensities higher than 20% were considered. While comparing treated to control profiles, only changes observed in all the three replicates of the treated plants were considered. The GTS was calculated for each treatment and expressed as a percentage of the non-variable bands compared to the total band number. The entire procedure above described was repeated for mature leaves (three 30 day leaves for each treatment) to check a possible recovery of the genotoxic damage.

### Data analysis

Normality and homogeneity of the variance of the data were assessed by the Shapiro–Wilk and Levene test, respectively. Data were analyzed with one-way ANOVA and, for count data, with Generalized Linear Model with a Poisson distribution, using IBM SPSS Statistics for Windows (IBM Corp. Released 2020, Version 27.0; IBM Corp, Armonk, NY, USA). Multiple comparison tests were performed with Tukey’s *post-hoc* test (*p* < 0.05).

## Results

### Analysis of morphological traits

The germination percentage was always 100% for the control, 1 and 10 Gy treatments. Morphological traits were differently affected by irradiation (Table 1); some traits were more susceptible than others to the treatments. Specifically, plant height did not differ between control and treated plants. Conversely, the number of the leaves and weight of the shoots increased at 1 Gy compared to control, although this increment was significant only for the shoots; the average shoot weight measured in these plants was indeed the highest (*i.e*., 12.8 g). Roots were significantly longer in plants grown from 10 Gy irradiated seeds, with an average length of 23.1 cm. Root weight was instead significantly lower in plants developed from irradiated seeds, with a decrease of about 33% observed in 1 Gy treated plants and even higher under 10 Gy treatment.

**Table 1:** Effects of X-ray irradiation on morphological traits of *Eruca sativa* plants.

	No. of leaves	Root length (cm)	Plant height (cm)	Shoot weight (g)	Root weight (g)
C	7.3 ± 1.9^ab^	10.1 ± 2.0^b^	15.2 ± 3.1	8.8 ± 0.7^b^	1.5 ± 0.4^a^
1 Gy	8.3 ± 1.8^a^	8.0 ± 2.1^b^	18.0 ± 3.3	12.8 ± 4.6^a^	1.0 ± 0.5^b^
10 Gy	5.4 ± 2.1^b^	23.1 ± 9.8^a^	16.1 ± 3.8	7.9 ± 1.9^b^	0.8 ± 0.5^b^
One-way ANOVA/GLM results	$\chi^2$ = 3.88/*p* = 0.048	F = 19.46/*p* = 6 × 10^−6^	F = 1.71/*p* = 0.20	F = 7.6/*p* = 0.002	F = 7.1/*p* = 0.003

**Note:**

Each value represents the mean ± SD, *n* = 10. Different letters indicate significant differences among the treatments according to Tukey’s *post-hoc* test with significant *p* < 0.05. C, control; 1 Gy and 10 Gy, treated plants.

### Stomata counting and surface

**Figure 1:**
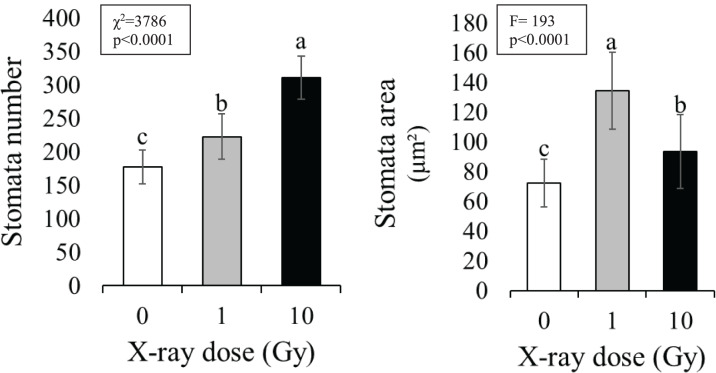
Mean ± SD of stomata number (*n* = 10 areas) and area (*n* = 100) in control and treated plants. Different letters indicate significant differences among treatments according to Tukey’s *post-hoc* test (*p* < 0.05).

**Figure 2:**
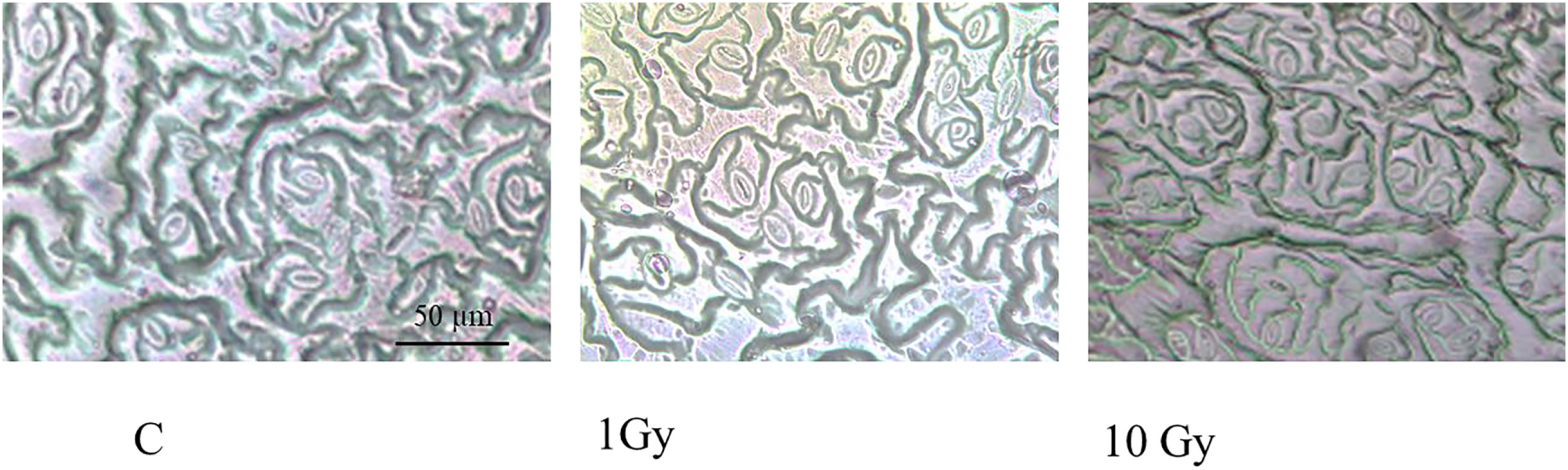
Leaf epidermis with stomata, in control (C) and and treated plants (1 and 10 Gy).

Stomata number (Fig. 1) significantly increased under IR stress compared to the control plants from an average of 177 for control plants to 313 in plants treated with 10 Gy, at parity of surface analyzed (160,000 µm^2^). A significant increase in stomal surface compared to control was also observed in plants treated with 1 and 10 Gy X-rays; particularly, stomal surface about doubled in 1 Gy treated plants, whereas it increased of 30% in 10 Gy treated plants. Hence,, this increment, also evident by observing the images under the light microscope (Fig. 2), was not dose-dependent but followed a hormesis trend (Fig. 1).

### Pigment content

**Figure 3:**
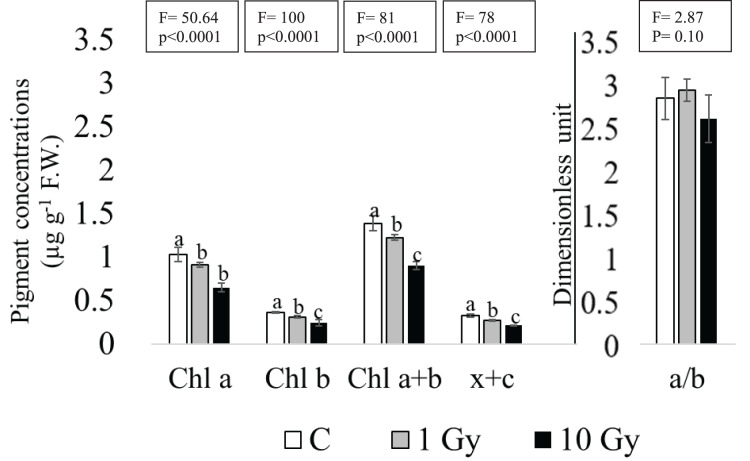
Mean ± SD (*n* = 5) of pigment contents in control and treated plants. Different letters indicate significant differences among the treatments according to Tukey’s *post-hoc* test with significant *p* < 0.05.

Although the absence of chlorosis and necrosis in the leaves of the plants from irradiated seeds, a dose dependent decrease was found for Chl contents, statistically significant at the highest dose of 10 Gy (Fig. 3). In fact, in these leaves Chl a decreased more than 30% compared to control (from 1.03 to 0.65 µg g^−1^ F.W.). Similarly, a significant decrease of Chl b was observed under both treatments, especially in leaves from 10 Gy treated samples. Total chlorophyll content (Chl a+b) enforced this trend, showing a significant decrease in both treatments compared to control. The Chl a/Chal b ratio (Fig. 3) was not significantly different among samples, probably due to the high variability observed in 10 Gy leaves. The sum of xanthophyll and carotenoids (x+c) showed a dose dependent statistically significant decrease from control to 10 Gy dose, as well.

### Protein analysis

**Figure 4:**
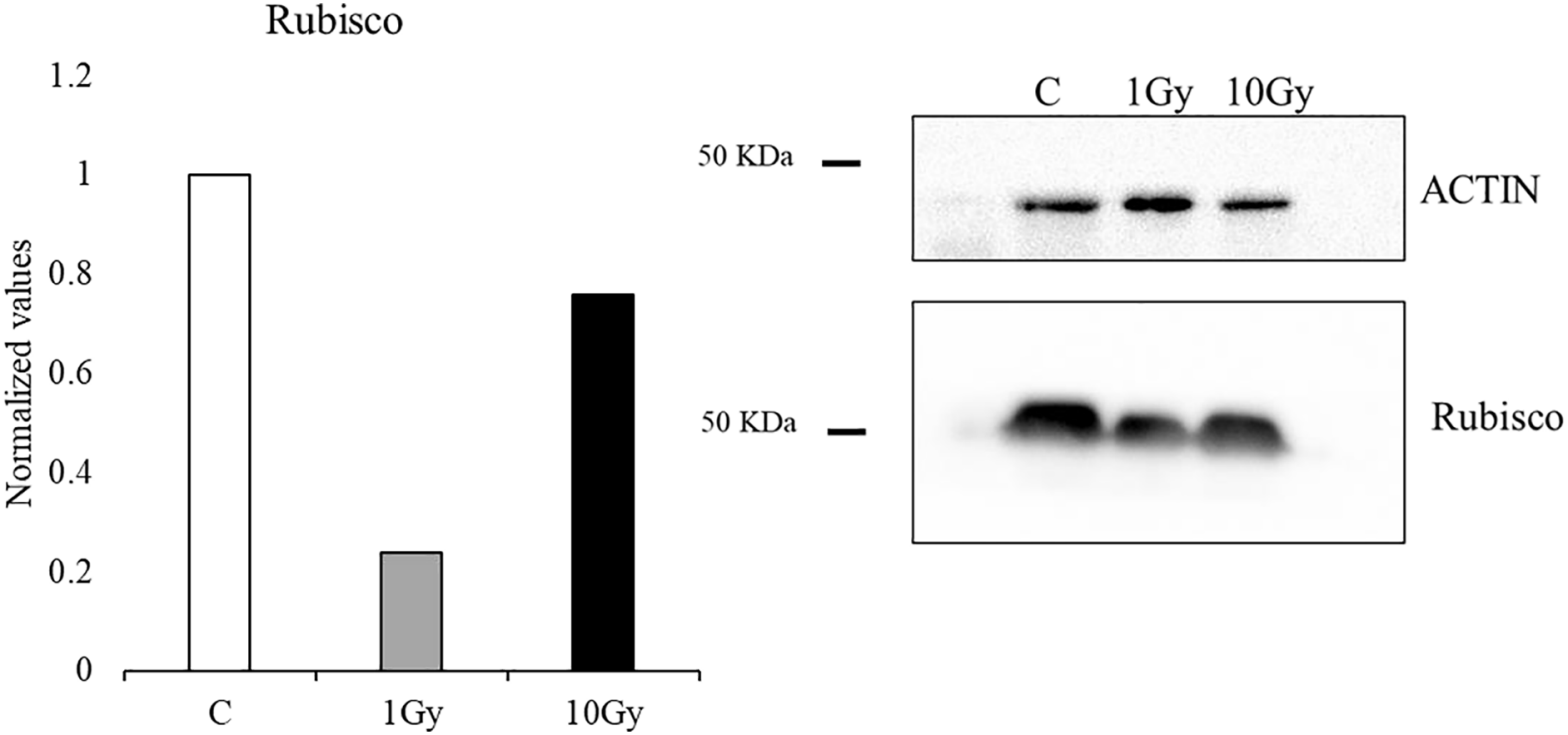
Western blot and densitometric analysis of Rubisco protein in control C and treated plants (1 and 10 Gy). Bar diagrams = pixel volumes of Rubisco.

Protein analysis of the leaves showed a reduction of the Rubisco concentration with both tested doses, compared to control plants, whose content was arbitrary set equal to 1. However, the decrease was more pronounced at 1 Gy, with a concentration of Rubisco about 24% of the control, while at 10 Gy a concentration of Rubisco of about 76% compared to the control plants was measured (Fig. 4).

### Phenols and total antioxidant content

**Figure 5:**
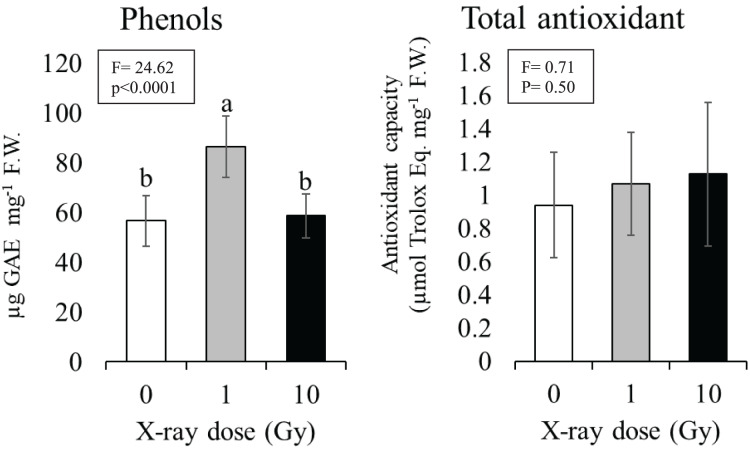
Mean ± SD (*n* = 10) of phenols and total antioxidant contents in control and treated plants. Different letters indicate significant differences according to Tukey’s *post-hoc* test with significant *p* < 0.05.

The 1 Gy dose determined a significant increase in phenol content of about 30% compared to control plants (from 57 to 86 µg) gallic acid equivalent per mg tissue (F.W.), while at 10 Gy dose phenol content resulted comparable to control (Fig. 5). Although an increasing trend was observed with the increasing dose no statistically significant differences were found among the total antioxidant contents of control plants compared to plants grown from seeds exposed to ionizing radiations; total antioxidant contents ranged from 0.93 (control) to 1.12 (10 Gy) µmol Trolox equivalent mg^−1^ F.W. (Fig. 5).

### Genotoxicity

A total of 48 reproducible bands were found in the control 15 d and 30 d leaves. The genome template stability (GTS) percentage (Table 2; Fig. S2) calculated in the control plants was 91%, which means that the 9% of the examined bands showed variations, mostly in form of increased/decreased band intensity (*i.e*., variation of type c and d). X-rays induced a noticeable decrease of the GTS percentage that halved under 1 Gy treatment and even more under 10 Gy, in the 15 d-leaves from irradiated seeds. In 1 Gy treated samples the higher contribution to the reduction of the GTS was given by the appearance and disappearance of bands (about 30%); whereas, similarly to control, in the 10 Gy treated samples most decrease of the GTS depended on the variation in band intensity (about 35%). In 30 d-leaves a partial recovery of the GTS percentages was observed in plants from irradiated seeds, more pronounced in 1 Gy plants, with a GTS reaching 69% and 56% in 1 and 10 Gy plants, respectively (Table 2).

**Table 2:** Average GTS percentage (*n* = 3) observed in leaves of control and treated samples.

GTS (%) in 15 d leaves				GTS (%) in 30 d leaves		
	a+b*	a+b+c+d			a+b	a+b+c+d
C	99	91		C	97	92
1 Gy	71	50		1 Gy	80	69
10 Gy	81	45		10 Gy	73	56

**Note:**

* a, new appeared bands; b, disappeared bands; c, bands showing higher intensity; d, bands showing lower intensity, in comparison with the banding profile of the control reference plant.

## Discussion

This work focused on the growth and physiological responses of *E. sativa* individuals grown from seeds irradiated by low (1 Gy) and high (10 Gy) X-ray doses. Our results indicated that IR influenced modifications of morpho-physiological traits. In a recent review, Gudkov et al. (2019) reported that the exposure to low doses of IR promoted the increase of some traits such as plant weight in *Triticum sp*. (Singh et al., 2013) or root weight in *Arabidopsis thaliana* L. (Vanhoudt et al., 2014), suggesting that hormesis could be a sustainable option to explain plant response under IR stress. Accordingly, in our study shoot weight and the number of the leaves followed this trend, being significantly higher in plants from 1 Gy irradiated seeds. Indeed, the 10 Gy treatment induced a significant root elongation, while the same treatment did not increase the shoot weight, which resulted significantly increased only in plants grown from 1 Gy treated seeds. In accordance with previous work, a stronger response of the roots respect to the shoots can be observed when the two organs are treated with the same IR dose (*e.g*., Beyaz et al., 2016; Desai & Rao, 2014); this behaviour could be due to the lower antioxidant content of the roots compared to the shoot (Gudkov et al., 2019). De Micco et al. (2014) found that IR irradiated seeds of *Solanum lycopersicum* L. produced plants with a higher number of larger leaves. In contrast, our results evidenced no clear trend in the production of leaves and a quite stable plant dimension (weight and height). It is worth noting that the irradiation dose range used by De Micco et al. (2014) was wider (0 to 100 Gy) and we cannot exclude other effects on *E. sativa* induced by doses higher than 10 Gy. Concerning morphological traits, it is noticeable that in plants from irradiated seeds these parameters showed a wide variation compared to control plants. These effects could be related to a decrease in plant vigor (*i.e*., homogeneity of the traits) induced by radiation, as already observed for the same species on board of the International Space Station (Chandler et al., 2020; Dowlath et al., 2021). It is well known that stomata vary in number and size, but also in their closing-opening cycle in response to several abiotic stresses (Lawson et al., 2014; Pääkkönen et al., 1998); effects caused by IR on stomata modulation are reported in the literature as well. For instance, Singh et al. (2013) evidenced a significant dose-dependent reduction of stomata size in *Psidium guajava* L. ex-posed to IR. Similarly, seedlings of mung bean (*Vigna radiata* L.), exposed to medium to high doses of IR, reduced the number of stomata with the increase of IR dose (De Micco et al., 2021). In mango leaves instead, stomata number remained unchanged following IR treatment (Indriyani, 2012). It is also reported that, under abiotic stress, an increase in stomata number can help support photosynthesis and can occur sometimes combined to a decrease of the stomata area (Sorrentino et al., 2021; Stolarska et al., 2007; Weryszko-Chmielewska & Chwil, 2005). In our experiment, we found a significant dose-dependent increase of the stomata number and a significant increase of the stomata areas higher at 1 Gy. We hypothesize that the lower dose could determine a non-specific response affecting both the number and the size of stomata. In fact, the increase in size could be regarded as a non-effective response to improve photosynthetic performance implying a longer time to induce turgor variation of the guard cells (Sorrentino et al., 2021). On the contrary, a more specific response, only based on the modulation of stomata number could occur under a higher stress (*i.e*., 10 Gy), ensuring a better photosynthetic performance. This interpretation of the results is enforced by the pattern of the Rubisco, which drastically decreased at 1 Gy and partly recovered at 10 Gy. Nevertheless, a decrease of pigment content was observed under IR, in line with other literature reports (Alikamanoglu, Yaycili & Sen, 2011; De Micco et al., 2014; Goh et al., 2014), suggesting that this parameter could be used as an efficient marker of IR stress thanks to its univocal pattern regardless other morpho-physiological traits (Kovacs & Keresztes, 2002; Olsen & Dineva, 2017).

A decline in chlorophyll content could be induced by a reduced expression of the related genes (Kim et al., 2009), or the oxidation of the pigments mediated by the ROS (Dartnell et al., 2011), or disarrangements of the antenna complex. However, the latter condition is often associated to an increase in the chlorophyll a/b ratio (*e.g*., Jia & Li, 2008; Macovei et al., 2014; Marcu, Cristea & Daraban, 2013). In *Eruca* plants from irradiated seeds chlorophyll a/b ratio was not significantly different from control plants, so we cannot support the same conclusions. Carotenoids are involved in photosynthesis and in photoprotection of photosystem II (PSII), these pigments protect plant cells against UV-B and IR (Kovacs & Keresztes, 2002). According to literature, the carotenoid contents typically change under IR stress (Gudkov et al., 2019); however, this response is often dose and specie specific (*e.g*., Arena, De Micco & De Maio, 2014; Choudhary & Agrawal, 2014; Esnault, Legue & Chenal, 2010). We found a dose dependent reduction of these pigments as previously highlighted by Kim et al. (2009). Antioxidant compounds play an important role in defending plants from reactive oxygen species (ROS) produced consequently to the exposure to ionizing radiation (Sharma et al., 2012). In general, under IR stress an increase in antioxidant compounds was observed, with a dose-dependent trend (*e.g*., Bhat, Sridhar & Tomita-Yokotani, 2007; Štajner, Milošević & Popović, 2007). Some authors found than this increase could be related to an increase of phenylalanine ammonia-lyase, which is responsible of the synthesis of phenolic compounds (*e.g*., Bhat, Sridhar & Tomita-Yokotani, 2007; Oufedjikh et al., 2000). Other authors (Siddhuraju et al., 2002) hypothesized higher extractability of antioxidants due to the depolymerization of cell wall polysaccharides occurring under irradiation. Our results, in line with those reported for soybean (Dixit et al., 2010) highlighted a significant increase of phenols at the lower dose and a decrease or a return to control situation at the higher dose. Therefore, the lower doses seem to activate compensative processes that lead to the production of higher levels of antioxidant compounds, phenols in our case. To our knowledge, this is the first contribution investigating the genotoxic damage induced by IR, highlighting the occurrence of a recovery in plants from irradiated seeds. The multilocus approach by neutral molecular markers, as ISSR, allows to quantify the genotoxicity and the possible recovery, by calculating the genome template stability. As sessile organisms, plants are especially exposed to environmental hazards, included those harmful for DNA (Gimenez & Manzano-Agugliaro, 2017). Therefore, the set-up of mechanisms to reveal and repair DNA damages is essential to ensure the correction of DNA alterations to recover the original genetic information (Bray & West, 2005). In plants, the impairment of DNA damage repair mechanisms modifies cellular physio-logical processes such as the cell cycle, transcription and protein synthesis, also altering the normal development and growth pattern (Britt, 1999; Polyn, Willems & De Veylder, 2015). Specifically, it is reported in the literature that strand-breaks and base modifications induced by X-rays in plants, can be re-paired by homologous recombination and non-homologous end-joining with mechanisms mainly shared among all eukaryotes (Kim et al., 2019).

Our results showed that IR did determine a severe decrease in the genome template stability; however, a certain recovery was observed after two weeks, especially under the lowest dose (*i.e*., 1 Gy), suggesting that DNA damage detection and repair mechanisms are active. Although there is no published work on the timing of repair of genotoxic damage in plants, times of 48 h for repair of transcribed genes and several weeks for non-transcribed sequences are reported for animals (American Society for Biochemistry & Molecular Biology, 2019).

## Conclusions

X-ray treatments in *Eruca sativa* modified several morpho-physiological traits, although some features did not show a dose-dependent pattern. For most of the traits investigated, such as morphological characteristics and the antioxidant power, the lowest dose of 1 Gy seems to stimulate compensative responses to counteract the adverse effects caused by X-rays, according to a hormesis model. A feasible explanation of the high variability observed in treated plants for most considered traits could be the decrease of vigor induced by IR. The parameters examined related to photosynthesis, *i.e*., pigment content, stomata number and surface, and Rubisco concentration, taken together, seem to suggest a positive response of treated plants to IR; in fact, the decrease in pigment concentrations, was coupled to a significant increase of stomata number and, at least at the highest dose, a recovery of the Rubisco concentration, in the absence of necrosis and chlorosis. We considered genotoxicity under IR stress and its recovery in *E. sativa*. The results showed a severe genotoxic effect induced by IR, with a consequent reduction of the GTS, but also a recovery capacity of the treated plants, with an increase of the GTS, after 30 days from the sowing. In future, the use of more checkpoints for analyses and more doses over a wider range, as well as the focus on different metabolites, could help elucidate plant response in terms of morpho-physiological changes. Finally, the pigment content and genotoxic damage, showing univocal dose-dependent trends could be useful proxies in the evaluation of plant response to X-ray stress.

## Supplemental Information

10.7717/peerj.15281/supp-1 Supplemental Information 1 Raw data.

10.7717/peerj.15281/supp-2 Supplemental Information 2 DNA banding profiles from leaves of control (C) and treated (1 Gy and 10 Gy) plants amplified with ISSR 10 primer and analyzed by GelAnalyzer 2019 software for band counting and intensity assignment. Lane 1: 100 bp ladder.

## Additional Information and Declarations

### Data Availability

The following information was supplied regarding data availability: The raw measurements are available in the Supplemental File.
