# Supplementary figures and images for: Evaluation of morpho-physiological responses and genotoxicity in Eruca sativa (Mill.) grown in hydroponics from seeds exposed to X-rays
Source: PeerJ. 2023 Apr 26;11:e15281. doi: 10.7717/peerj.15281 (PMC10148638; doi:10.7717/peerj.15281)

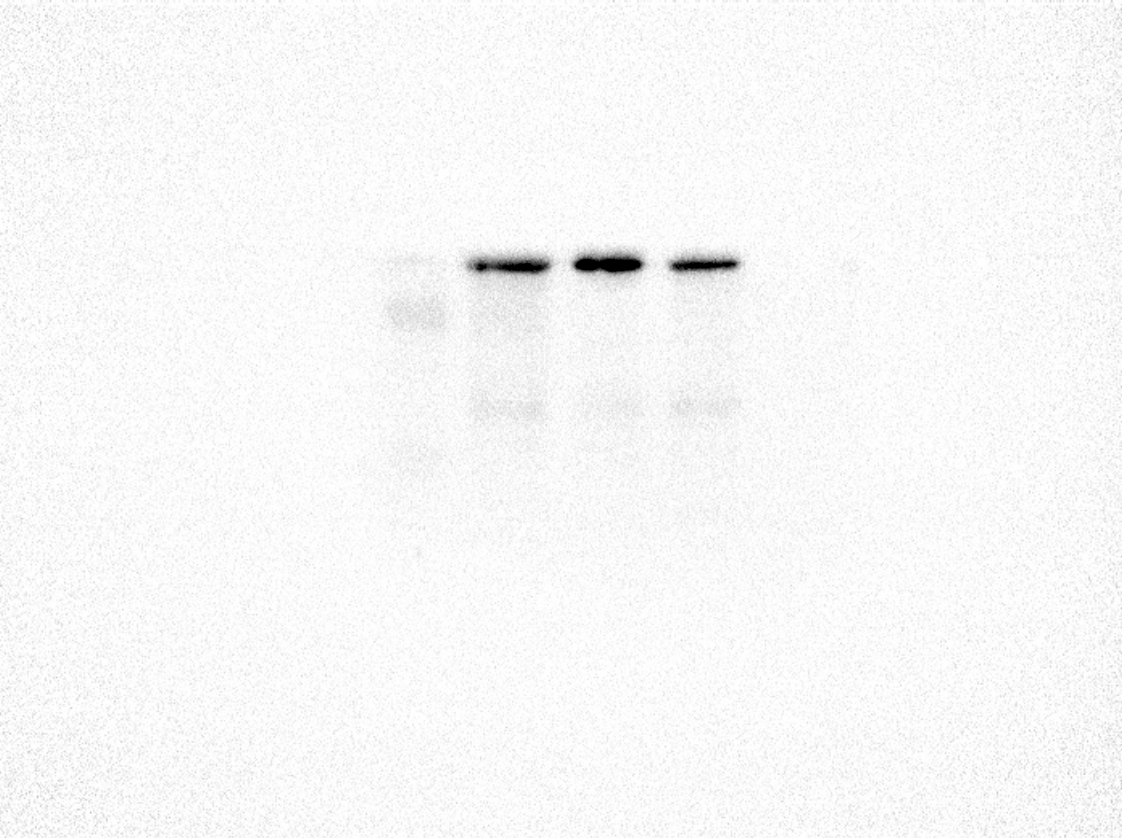

Supplement: Supplemental Information 1 [file peerj-11-15281-s001.zip › RAW DATA/actin.jpg]

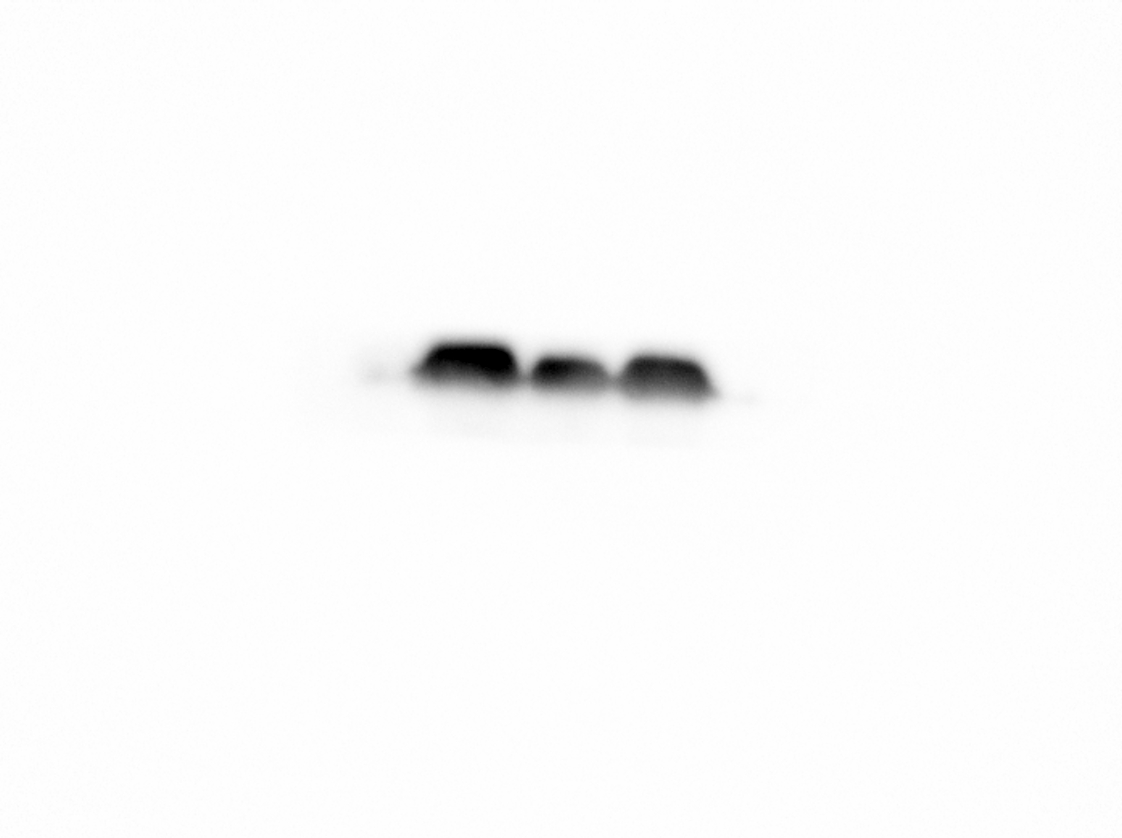

Supplement: Supplemental Information 1 [file peerj-11-15281-s001.zip › RAW DATA/RUBisco.jpg]
