# Supplementary material for: Evaluation of morpho-physiological responses and genotoxicity in Eruca sativa (Mill.) grown in hydroponics from seeds exposed to X-rays
Source: PeerJ. 2023 Apr 26;11:e15281. doi: 10.7717/peerj.15281 (PMC10148638; doi:10.7717/peerj.15281)
Supplement: Supplemental Information 2 — Lane 1: 100 bp ladder. [file peerj-11-15281-s002.zip › Supplementary/Figure S1.pdf]

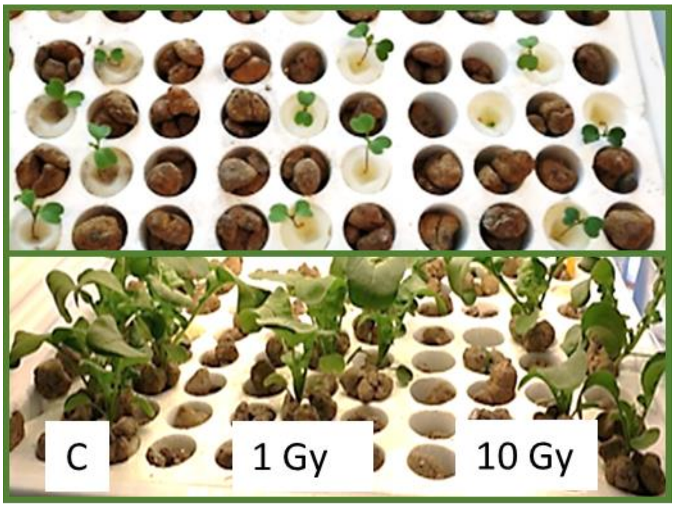

Figure S1. *Eruca sativa* from control seeds (C) and irradiated seeds (1 Gy and 10 Gy). Ten days (above) and 21-days old plantlets (below).
