# Supplementary material for: Evaluation of morpho-physiological responses and genotoxicity in Eruca sativa (Mill.) grown in hydroponics from seeds exposed to X-rays
Source: PeerJ. 2023 Apr 26;11:e15281. doi: 10.7717/peerj.15281 (PMC10148638; doi:10.7717/peerj.15281)
Supplement: Supplemental Information 2 — Lane 1: 100 bp ladder. [file peerj-11-15281-s002.zip › Supplementary/Figure S2.pdf]

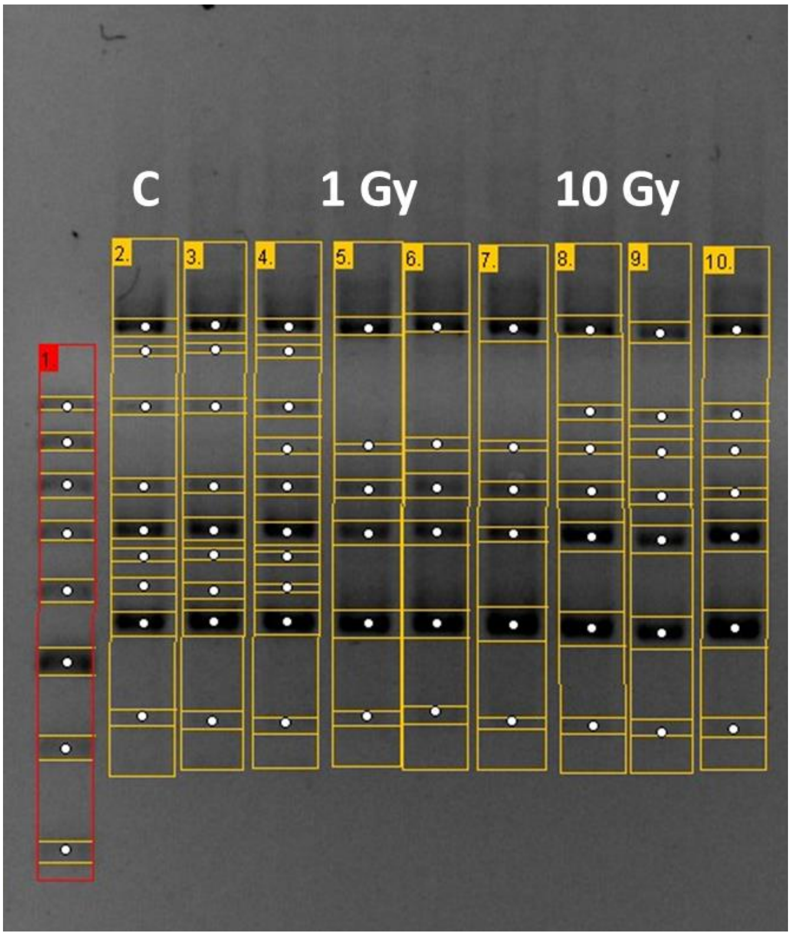

Figure S1. DNA banding profiles from leaves of control (C) and treated (1 Gy and 10 Gy) plants amplified with ISSR 10 primer and analyzed by GelAnalyzer 2019 software for band counting and intensity assignment. Lane 1: 100 bp ladder. See the text for further details.
