# Supplementary material for: Evaluation of morpho-physiological responses and genotoxicity in Eruca sativa (Mill.) grown in hydroponics from seeds exposed to X-rays
Source: PeerJ. 2023 Apr 26;11:e15281. doi: 10.7717/peerj.15281 (PMC10148638; doi:10.7717/peerj.15281)
Supplement: Supplemental Information 2 — Lane 1: 100 bp ladder. [file peerj-11-15281-s002.zip › Supplementary/Table S1.docx]

**Table S1. Details of the six primers used and melting temperatures (Tm).**

**Primer code Primer sequence 5'-3' Number of bases Tm(°C)**

1. 10 AGAGAGAGAGAGAGYC 16 48
2. W814 CTCTCTCTCTCTCTCTTG 18 48
3. TE GTGGTGGTGGTGRC 14 44
4. HAD CTCCTCCTCCTCRC 14 44
5. MAN CACCACCACCACRC 14 44
6. DAT GAGAGAGAGAGAGARC 16 46
